# Impact of the nursing work environment on patient satisfaction with care: A cluster-randomised organisational intervention

**DOI:** 10.1016/j.ijnsa.2026.100606

**Published:** 2026-06-30

**Authors:** Diego Montano

**Affiliations:** Research Group of Medical Sociology, Institute of the History, Philosophy and Ethics of Medicine, University of Ulm, Oberberghof 7 89081, Ulm, Germany

**Keywords:** Quality of care, Psychosocial workload in hospital wards, Patient outcomes, Inpatient health care

## Abstract

**Objective:**

To investigate the hypothesis that an organisational intervention with health care workers would help improve patient satisfaction with care.

**Methods:**

An organisational intervention with health care workers was implemented in 10 German hospitals. Wards were randomised either to the intervention or control arm. The intervention was participative and aimed to reduce the psychosocial workload, and improve the work environment and job performance of health care workers. It was hypothesised that patient satisfaction with care would also improve as a result of the intervention. A total of 1,664 patients from several wards in the participating hospitals were interviewed before and after the intervention. The research hypotheses were investigated with cumulative logit regression models on four dimensions of patient satisfaction, namely, trust in health care workers, received support, availability of health care workers and patient decisional control.

**Results:**

The analyses did not provide evidence that the intervention had a positive impact on patient trust (OR 1.21, 95% CI [0.79–1.85]), support (OR 1.28, 95% CI [0.82–1.99]), availability of health workers (OR 1.13, 95% CI [0.75–1.69]) and patient decisional control (OR 1.01, 95% CI [0.68–1.48]). On the contrary, the largest proportion of explained variance of the scales was related to education and self-rated health.

**Conclusions:**

Individual patient characteristics were found to be the most influential factors of patient satisfaction appraisals. More work seems to be required to understand the mechanisms linking the nursing work environment and patient outcomes in order to inform effective organisational interventions.

Registration number DRKS00021138, German Registry of Clinical Studies.

## Highlights

• **What is already known**
• Patient satisfaction with care depends on individual and situational factors.
• The psychosocial workload of health care workers affects their job performance.
• **What this paper adds**
• Patient satisfaction with care depends largely on education and subjective health.
• The psychosocial workload of workers is less related to patients’ appraisals of care.
• Situational features or events may be more suitable indicators of quality of care.

## 1 Introduction

From an occupational point of view the nature of nursing work consists of the interactions that health care workers and patients sustain in the course of health services provision (Hacker, 2009). Nursing tasks are performed on patients who, at the same time, are required to cooperate with health care workers so that they can co-create the services being provided to them (Hacker, 2009). The concept of co-creation means that the recipients of a service (patients) are required to regulate their own behaviour, emotions and intentions and to cooperate with the services providers (health care workers) in order to instantiate the services themselves (health care) (Hacker, 2009). From this perspective, the provision of services is unique in comparison to other types of labour, e.g., in the manufacturing industry in which the objects of work tasks (for instance, raw materials) lack own agency. Usually, health care services correspond to highly complex and demanding work routines involving high task interdependence, interdisciplinary work, inflexible work schedules, high responsibility for patient’s safety, and increased cognitive and emotional demands (Institute of Medicine (US). Committee on the Work Environment for Nurses and Patient Safety, 2004).

Patient-related outcomes result not only from the effectiveness and adequate implementation of the particular medical treatments and the individual regeneration capacity of the organism, but also from the manner how health care providers determine their internal work processes and the working conditions of the nursing staff. For instance, previous findings have reported lower patient mortality in hospitals with higher nurse staffing and improved nurse work environment (Aiken et al., 2011). Lower patient-related adverse events rates have been observed in hospitals with higher nursing care hours per day and more positive perceptions of the nursing practice environment (Dubois et al., 2013). In acute specialist units such as intensive therapy, a higher proportion of nurses assigned to patients was associated to lower inpatient mortality rates (Driscoll et al., 2017). Nurses working hours exceeding 12 h per shift and 40 h per week seem to increase the risk of adverse events among patients (Di Muzio et al., 2019). Moreover, the working conditions of health care workers may affect not only the workers themselves, but patient outcomes as well. For instance, previous meta-analytic findings have indicated that the risk of medication errors and patient-related adverse effects may increase as a result of higher burnout rates among health care workers (Li et al., 2024a).

Accordingly, it has long been recognised that patient outcomes may benefit from improvements of the nursing work environment related to leadership, nursing scheduled shifts, overtime restrictions, improved definition of work tasks and the use of certain tools or technologies (Institute of Medicine (US). Committee on the Work Environment for Nurses and Patient Safety, 2004). In fact, in countries such as the United States or Germany, patient satisfaction scales have become mandatory in the quality assurance guidelines of the corresponding health care systems (e.g., the Patient Experience of Care domain in the US – Federal Register, 2012, Vol. 77, No. 170; or the Quality Management Requirements of the German Statutory Health Insurance System - Bundesanzeiger AT 19.04.2024 B5). It is implicitly assumed that patient satisfaction with care, as an indicator of perceived performance (Johnson and Fornell, 1991), strongly correlates with the quality of health care services. Previous research has identified several aspects of the nursing work environment that may be causally related to patient satisfaction such as the patient-to-nurse ratio (Aiken et al., 2018), communication and nursing skills and nurse availability (Otani and Kurz, 2004), nursing leadership (Wong et al., 2013), and care left undone, number of items of missed care and rationing of caring (Recio-Saucedo et al., 2017).

Notwithstanding the indications that the work environment may have a substantial impact on patient outcomes and patient satisfaction with care, it is still unknown what health care businesses and professionals may actually do to improve patient satisfaction. Some cross-sectional studies have investigated the associations between certain aspects of the work environment and patients satisfaction including nurse participation in hospital affairs, nursing foundations for quality care, nurse manager ability, leadership and support of nurses, staffing and resource adequacy and collegial nurse-physician relationships (Lake et al., 2019, Simonetti et al., 2023, You et al., 2013). However, these studies focus on a broad categorisation of “good” vs. “poor” work environment (based on the quantiles of the distribution) and patient satisfaction, rather than on each specific aspect of the work environment. Hence, it seems that the knowledge on potential causal mechanisms relating the nursing work environment and patient satisfaction with care are still largely unknown. Consequently, it is still uncertain what types of organisational interventions may be more effective to improve the provision of care. Against this background, the present study contributes to previous research by examining how the modification of specific aspects of the nursing work environment may contribute to a higher patient satisfaction with care. In contrast to previous interventions with health care workers which lacked a randomised design and did not address directly the nursing work environment (Li et al., 2024b), the present study is a cluster-randomised intervention that addressed explicitly the physical and psychosocial workload of health care workers in order to improve patients satisfaction with care.

## 2 Methods

### 2.1 The study

The intervention study “HALTgeben” (“Higher Patient Satisfaction through Fair Working Conditions in Health Care”) was a two-arm cluster-randomised intervention with health care workers in German hospitals conducted from 2019 to 2021 (Registration trial DRKS00021138 on the German Registry of Clinical Studies). The units of randomisation were clusters of the hospital wards of the health care workers participating in the study that had the same specialisation and similar organisational structure, e.g., anaesthesia, surgery, paediatrics, cardiology, etc. The primary outcomes of the intervention were the self-assessed physical and mental work ability of health care workers. The secondary outcome was patient satisfaction with care. The study protocol with detailed information on randomisation, inclusion and exclusion criteria, and power analysis for the main outcomes has already been reported elsewhere (Montano et al., 2020). In the present investigation the results concerning the secondary outcome, i.e., patient satisfaction with care, are reported. The quantitative evaluation of the intervention was conducted by a group of researchers who were independent from the teams who conceived and carried out the intervention on the field.

### 2.2 The intervention

The organisational intervention aimed to change the working conditions of health care workers in the wards by recurring to the concept of work ability, which states that a person’s work ability is the ability to perform some kind of work given a person’s set of basic competences and occupational skills (Tengland, 2011). The intervention sought to align the individual characteristics and capacities of health care workers with the work and task processes throughout the different life phases (e.g., living single, family formation). The intervention was conducted in four phases. First, the consultants assessed the organisational structure of the wards participating in the study according to key organisational features such as work task definition, shift schedules or operating procedures. Second, the consultants conducted semi-structured interviews with voluntary employees and supervisors on topics such as work organisation and processes, works tasks and workload. Third, in a series of workshops conducted by the consultants who conceived and carried out the intervention, health care workers proposed measures that were meant to improve their own working conditions. And fourth, the decision to implement or reject the proposed measures was taken by task groups composed of managing board executives, managers of the health care departments, works council representatives, quality management and human resources representatives.

### 2.3 Research hypotheses

**Fig. 1:**
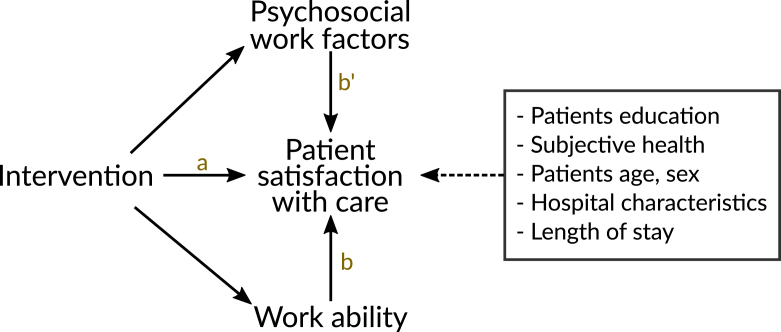
Graph of the assumed causal process in the statistical models. Dotted lines indicate potential confounding effects.

It was hypothesised that the measures proposed and implemented in the wards would improve the work environment in areas such as the coordination of work, the alignment of individual capacities and work demands, the definition of work tasks and processes, and reduce the psychosocial workload. Consequently, on the basis of the associations discussed in the introduction section between the work environment of health care workers and patient-related outcomes, it was expected that:

Hypothesis 1 The changes in the work environment in the hospital wards would contribute to a higher job performance and, therefore, to increasing levels of patient satisfaction with care (path a in Fig. 1).

Given that the intervention with health care workers also addressed the psychosocial workload in the wards, it was expected that:

Hypothesis 2 The changes in physical and mental work ability and some key psychosocial work factors addressed in the intervention such as job stress, affective and cognitive demands would lead to increasing levels of patient satisfaction with care (paths b and $b^{\prime }$ in Fig. 1).

Given the fact that patient satisfaction has been found to depend also on different patient and hospital characteristics (Batbaatar et al., 2016), a relevant set of confounding variables were considered explicitly in the research hypotheses (Fig. 1). In addition, the intervention can be regarded as a social field experiment concerning patient satisfaction (Baldassarri and Abascal, 2017), so that it is possible to obtain robust estimates of the hypothesised intervention effects in naturally occurring health care settings due to the random assignment of wards to the study arms.

### 2.4 Data

**Health care workers data**. Health care workers participating in the study completed a questionnaire covering socio-demographic information, physical and psychosocial working conditions, work ability, and perceived physical and mental health at baseline and two follow-up times at approximately 6 and 12 months after the intervention. In the present study the following psychometric scales corresponding to the psychosocial work factors were included in the analyses: (1) emotional demands at work (3 items), (2) cognitive demands (5 items), (3) job stress as measured by the effort-reward imbalance (ERI) questionnaire (17 items), (4) overcommitment (6 items), and (6) the subjective physical and mental work ability of health care workers (2 items). The concepts of emotional and cognitive demands are based on the general notion of several stress theories positing that certain work situations and/or characteristics represent stressors directly related to emotional and cognitive responses of employees (Kristensen et al., 2005). The emotional and cognitive demands were measured with two scales of the German version of the Copenhagen Psychosocial Questionnaire (COPSOQ) (Nübling et al., 2005). The corresponding scales were calculated as average scores ranging from 1 to 5 (1: “never”, 2: “rarely”, 3: “sometimes”, 4: “often”, 5: “always”), with higher scores indicating higher cognitive and emotional demands. Emotional demands were measured by asking workers whether they felt emotionally involved in their job tasks or their job was emotionally distressing. Cognitive demands were measured by items asking the extent to which workers needed to cope with a large amount of information, pay constant attention to different tasks, memorise new tasks or routines, generate new ideas or take difficult decisions.

The ERI model in occupational health is based on three dimensions: work efforts, work rewards and overcommitment (Siegrist, 1996). The model posits that job stress is due to the perception of lack of reciprocity between work efforts and rewards. Overcommitment denotes the failure to withdraw from work duties and represents a health-adverse personal coping pattern. The ERI questionnaire consists of the three scales efforts, rewards and overcommitment. The average scores of the efforts and overcommitment scales ranged from 1 to 4, with higher scores indicating a higher expression of the trait. On the contrary, higher scores of the rewards scale indicate less perceived rewards (range 1 to 4). The Effort-Reward Imbalance scores are defined and calculated as the ratio of the average scores of the effort scale (6 items) and the reward scale (11 items), with higher scores indicating higher job stress. Reliability coefficients of the effort and reward scales in this study were 0.80 and 0.79, respectively. The physical and mental work ability were measured with two items of the Work Ability Index Questionnaire, namely, “How would you appraise your current work ability in relation to the physical [mental] work demands?” with answer format ranging from 1 to 5 (1: poor, 2: not good, 3. good, 4: very good and 5: excellent) (Hasselhorn and Freude, 2007). The data of health care workers used in the present study was averaged at the hospital and cluster level. The reliability of the average scores was assessed by calculating the intraclass correlation coefficient (ICC2) according to the formula of McGraw and Wong (Table 5, model 2 A) (McGraw and Wong, 1996). Following the results of Woehr et al. (2015), a ICC2 higher than 0.6 was considered as an indication of an adequate reliability of health care workers group means at the hospital and cluster level.

**Patients data**. Data on patients in the intervention and control wards were collected cross-sectionally at the same time as the data collection of health care workers proceeded in the years 2019, 2020 and 2021. The target sample size of patients in each survey wave was 600. Eligibility criteria for the patient surveys was being older than 18 years, being able to give informed consent to participation, being a responsive patient and having sufficient skills in German. After obtaining patients consent to study participation, the interviewers collected questionnaire data on socioeconomic information (age, sex, education), a single item on general subjective health (from 1: poor to 5: very good), length of stay in the hospital (days), hospital type and two validated questionnaires of patient satisfaction with care, namely, the Cologne Patient Questionnaire (Pfaff et al., 2001) and the Individualised Care Scale (Köberich et al., 2015). Education comprised high (secondary) education, low (secondary) education, vocational education and other education including mostly older secondary educational titles or just primary education.

The Cologne Patient Questionnaire comprises three scales: trust in health care workers, support and availability of health care workers (Pfaff et al., 2001). Trust is conceptualised as the expectation that health care workers will take responsibility for patients. The trust scale comprised items covering honest and open communication of health care workers, the possibility of patients to express their thoughts, and the perceived skills of health care workers. Support and availability refer to specific features of the social relationship between health care workers and patients which may foster timely and appropriate care for patients concerns and needs. The support scale addressed behaviours such as perceived support to deal with patients’ health complaints, readiness to help and health care workers dependability. The availability scale comprised items related to whether health care workers were available at the wards to address the questions and needs of patients and their relatives.

The trust, support and availability scales consisted of 5, 3 and 4 items, respectively. All items were rated on a 4-point scale ranging from 1: “strongly disagree”, 2: “disagree”, 3: “agree” to 4: “strongly agree”. Thus, the average scores ranged from 1 to 4 with higher values indicating a higher level on the latent construct, e.g., higher trust in health care workers. Due to the fact that about 44% of patients did not report having their relatives at the hospital, the item related to patients’ relatives was excluded from the scoring of the availability scale.

The scale of patients decisional control over care was taken from the questionnaire Individualised Care Scale. Patients decisional control is defined as patients’ perceptions of how they may exert influence over their care (Suhonen et al., 2005). The scale of patients decisional control comprises 6 items that assess the degree to which patients wishes and needs were taken into account during their stay in the wards (i.e., need of information on one’s own health and consideration of one’s own opinions and decisions regarding personal care). The scale was rated on a 5-point scale ranging from 1: “strongly disagree”, 2: “disagree”, 3: “neither nor”, 4: “agree” to 5: “strongly agree”. Hence, average scores ranged from 1 to 5 with higher values indicating a higher level on the latent construct. Patient satisfaction with care represents in this study patients appraisals of key features of the relationship with health care workers and their decision latitude over their own care.

The response rate of contacted patients was 59.2%. The patient survey was conducted in the wards where the participant health care workers were usually deployed. Due to clinical and patient safety reasons some wards were excluded from the patient surveys, e.g., intensive care units and anaesthesia wards. The surveys were conducted in the following wards of the 10 hospitals that took part in the study: geriatrics, cardiology, gynaecology, internal medicine, urology, surgery, gastroenterology, ophthalmology, pulmonology, psychiatry and otorhinolaryngology.

### 2.5 Statistical analysis

The presence of ceiling effects of the scales of patient satisfaction with care were assessed by estimating the percentage of scores attaining the corresponding scale maximum (McHorney and Tarlov, 1995). Due to the fact that patient satisfaction scales are usually highly skewed (Schoenfelder et al., 2011), cumulative logit regression models were estimated. These type of models require only that the dependent variables represent ordinal measurements (Fahrmeir et al., 2013), as it is the case for the scales of patient satisfaction. The cumulative logit models estimate the probability that the dependent variable Y takes a value larger than a certain observed response r, i.e., P(Y>r). The regression equation is obtained by defining a latent variable $u=X\beta^{T}+\epsilon$ with residual ϵ, which is then plugged in a cumulative distribution function $P\left(Y>r\right)=F\left(\theta_{r}+X\beta^{T}\right)$, where $\theta_{r}$ corresponds to the threshold between adjacent values, say s>r, of the dependent variable Y. In the present study, the cumulative distribution function F(⋅) corresponds to the logistic distribution and, thus, the vector of coefficients $\beta^{T}$ can be expressed as the odds ratios of observing the next higher value of the dependent variable Y given a set of covariates X, i.e., the chances that respondents endorse higher answer categories in the patient satisfaction scales. Odds ratios greater than 1 indicate increasing patient satisfaction; values less than 1, on the contrary, less satisfaction. Even though the data were to some extent clustered due to the cluster-randomised design, the results of preliminary analyses comparing model fit between models with and without random effects did not indicate substantial improvements of model fit by using more complex models with random effects. Hence, only the fixed effects of the cumulative logit models were estimated and reported. As per study protocol (Montano et al., 2020), the mean scores of the psychometric scales were computed with available items if no more than 30% of the items defining the scale were missing. In addition, sensitivity analyses performed to assess the consistency of regression estimates with and without multiple imputation did not suggest large discrepancies of the corresponding estimates obtained with both datasets. Hence, the analyses were performed only on all available records following the intention-to-treat approach.

Hypothesis 1 was investigated with a cumulative logit regression (model 1) in which the direct intervention effects were estimated after controlling for socioeconomic characteristics of patients, hospital-related confounders and survey waves. Hypothesis 2 was tested with two cumulative logit regression models which included the ward-level means of the psychosocial workload and physical and mental work ability of health care workers. In model 2 the main effects of the psychosocial workload and the work ability of workers were estimated, whereas model 3 included the corresponding moderation effects of the intervention. According to the mediation conditions of Baron and Kenny (Baron and Kenny, 1986), the ward-level means of work ability and the psychosocial load were expected to be positively (negatively) associated with patient satisfaction with care, paths b and $b^{\prime }$ in Fig. 1, respectively. Thus, it was expected that the moderation effects of the intervention would result in increased patient satisfaction in the intervention wards, but not in the control ones. An interaction term of survey wave and intervention needed to be included in model 2 due to the fact that there were statistically significant differences in the scales of patient satisfaction with care between the intervention and control wards at baseline, even though the randomisation of workers was successful (Montano et al., 2020). The p-values and the significance level of the confidence intervals were adjusted by the method of Bonferroni (4 tests). The power analysis was based on the methods proposed by Whitehead for odds ratios of cumulative logit models (Whitehead, 1993).

The HALTgeben study was approved by the Ethics Committee of the University of Ulm under the registration number 99/19. All participants provided written informed consent prior to study enrolment.

## 3 Results

The sample was composed primarily of patients older than 50 years with vocational to low (secondary) education (Table 1a). The target sample size of 600 patients could be achieved at each follow-up time with the exception of the 2020 survey in which access restrictions to all health care facilities were imposed by the German government during the COVID-19 pandemic. The number of patients in the control and intervention wards were 1093 and 559 respectively. These samples sizes allow the estimation of effect sizes as small as d = 0.2 (i.e., approximately an odds ratio of 1.44 as explained in the discussion section below) between the control and intervention group with a power of 0.87, 0.83, 0.90 and 0.93 for the scales trust, support, availability and decisional control, respectively.

The majority of patients was interviewed in control wards and general hospitals due to the fact that these are larger and offer health services in all medical fields. Concerning the psychometric properties of the instruments, the reliability estimates of the scales of patient satisfaction with care were acceptable (Cronbach’s α> 0.7) (Table 1b). Large ceiling effects were observed, in particular for the trust and support scales, a fact that confirmed the adequacy of the cumulative logit regression instead of the linear regression models. Furthermore, the reliability of the ward-level means of the scales of the psychosocial work factors that were estimated with the health care workers dataset were also acceptable (ICC2 > 0.6), except for the cognitive demands scale whose ICC2 estimate was of a slightly lower magnitude. The ICC2 estimates support, in general, the use of the ward-level means in the statistical analysis as reliable estimates of the psychosocial workload in the wards.

Concerning the direct intervention effects on patients satisfaction with care (Hypothesis 1), the results of the cumulative logit regression (model 1) did not suggest that the intervention had any effect on the scales of patient satisfaction (Table 2). On the contrary, the largest associations were observed for patient characteristics. Individuals whose highest qualification was primary education or other types of secondary education tended to be more satisfied with care than patients with high secondary education. The smallest odds ratios were observed for patients in the psychiatric and geriatric hospitals who reported very low perceived trust in health care workers, support and availability, in comparison to patients interviewed in general hospitals. In addition, patients perceiving a better subjective health status were more satisfied in all dimensions of care, namely trust, perceived support and availability of health care workers and decisional control. The odds ratio estimates corresponding to each survey year suggest that patients in 2020 and 2021 were more satisfied with care than patients interviewed in 2019.

**Table 1a:** Descriptive statistics of the study population.

Categorical variables^a^			

**Variable**		%	**n**
Age	Age 18-49 [reference]	21.7	359
	Age 50–69	33.7	558
	Age 70 and older	44.7	740
Sex	Male [reference]	49.0	812
	Female	51.0	845
Education	High or tertiary education [reference]	16.7	274
	Low (secondary) education	30.0	493
	Vocational education	33.9	556
	Other education	19.4	319
Hospital type	General hospital [reference]	88.2	1468
	Psychiatric hospital	11.8	196
Study arms	Patients in control wards [reference]	66.2	1093
	Patients in intervention wards	33.8	559
Survey wave	Year 2019 [reference]	37.7	628
	Year 2020	23.5	391
	Year 2021	38.8	645
Total sample			1664

**Continuous variables**^b^			

**Variable**		**M**	**SD**
Length of stay		8.12	7.76
Patient subjective health		2.89	0.95

a Percent values (%) and frequencies (N).

b Means (M) and standard deviation (SD).

**Table 1b:** Means and standard deviations in parentheses of the psychometric scales. Cronbach’s alpha and intraclass correlation of reliability of average scores (ICC2).

Scales of patient satisfaction with care	

Trust in health care workers	3.80 (0.37), α = 0.85, ceiling: 62.9%
Support received by health care workers	3.75 (0.46), α = 0.87, ceiling: 69.5%
Availability of health care workers	3.73 (0.43), α = 0.78, ceiling: 56.8%
Decisional control of patients	4.40 (0.55), α = 0.71, ceiling: 25.0%

Scales of the psychosocial load of health care workers	

Emotional demands	4.30 (0.29), α = 0.73, ICC2 = 0.57
Cognitive demands	3.85 (0.42), α = 0.69, ICC2 = 0.56
Effort-reward imbalance	0.88 (0.24), ICC2 = 0.61
Overcommitment	2.73 (0.46), α = 0.79, ICC2 = 0.72
Physical work ability	2.97 (0.63), ICC2 = 0.63
Mental work ability	2.85 (0.61), ICC2 = 0.66

Concerning Hypothesis 2, the results did not provide support to the assumption that patient satisfaction with care improved as a result of the potential intervention effects on the physical and mental work ability of health care workers (Table 3). Contrary to the expected direction of associations (path b in Fig. 1), a higher perceived physical work ability at the ward level was associated with lower perceived availability and decisional control of patients (OR 0.71, 95% CI [0.55-0.91] and 0.78, 95% CI [0.62-0.98], respectively). Furthermore, the results concerning the direction of associations between the psychosocial workload and patient satisfaction were not consistent (path $b^{\prime }$ in Fig. 1). Higher emotional demands and a higher effort-reward imbalance in the wards were associated with a lower perceived availability of health care workers (OR 0.55, 95% CI [0.37-0.80] and 0.44, 95% CI [0.20-0.96], respectively). At the same time, however, a higher level of overcommitment was related to an increased perceived availability of health care workers (OR 1.93, 95% CI [1.19-3.14]).

**Table 2:** Results of the regression analysis concerning hypothesis 1.

Variable	Trust in health care workers		Support received by health care workers		Availability of health care workers		Decisional control of patients	
	OR	95% CI	OR	95% CI	OR	95% CI	OR	95% CI
Female (vs. male)	1.14	0.88–1.47	1.03	0.78–1.37	1.09	0.85–1.41	1.06	0.85-1.32
Age 50-69 (vs. 18-49)	1.10	0.77–1.58	1.19	0.80–1.76	1.11	0.77–1.58	0.97	0.71-1.34
Age 70 and older	1.23	0.84–1.80	1.20	0.79–1.81	1.10	0.76–1.60	0.76	0.55-1.06
Low (secondary) education (vs. high)	1.22	0.81–1.82	1.33	0.86–2.06	0.89	0.60–1.31	1.00	0.70-1.42
Vocational educational	1.12	0.77–1.63	1.13	0.76–1.69	1.06	0.74–1.53	1.13	0.81-1.57
Other education	1.54	0.98–2.44	1.86**	1.12–3.11	2.96***	1.83–4.78	1.14	0.77-1.69
Subjective health	1.31***	1.15–1.50	1.33***	1.15–1.54	1.24***	1.08–1.41	1.24***	1.10-1.39
Psychiatric hospital (vs. general hospital)	0.25***	0.16–0.39	0.28***	0.18–0.45	0.30***	0.19–0.46	0.75	0.50-1.13
Length of stay	0.99	0.98–1.01	1.01	0.99–1.03	1.01	0.99–1.03	1.01	0.99-1.03
Intervention ward (vs. control ward)	1.21	0.79–1.85	1.28	0.82–1.99	1.13	0.75–1.69	1.01	0.68-1.48
Survey wave 2020 (vs. 2019)	2.29***	1.50–3.50	2.58***	1.65–4.02	1.72**	1.17–2.52	1.38	0.96-1.98
Survey wave 2021	2.11***	1.48–3.02	3.63***	2.43–5.41	3.51***	2.45–5.03	1.21	0.89-1.66
Intervention ward × 2020 (vs. 2019)	0.66	0.32–1.35	0.85	0.39–1.86	0.80	0.42–1.55	1.00	0.54-1.84
Intervention ward × 2021 (vs. 2019)	0.86	0.46–1.60	0.75	0.37–1.50	1.53	0.78–2.97	1.07	0.63-1.83

Sample size	1,584		1,578		1,607		1,605	

p<0.001 “***”, p<0.01 “**” p<0.05 “*”. OR: odds ratio, 95% CI: 95% confidence interval. Subjective health was treated as a continuous variable with higher scores indicating better health (1 to 5) and odds ratio representing change in the outcomes per one-unit increase in subjective health. Non-significant interaction terms indicate that there is no additional explained variance beyond the main effects.

In general, the results of the moderation analysis reported in Table 4 did not indicate the presence of interaction effects between the work environment and the intervention. The odds ratio estimates did not reveal a distinct pattern of overall improvement of patient satisfaction in the intervention wards, but rather similar associations in both study arms. Moreover, the interaction effects of the effort-reward imbalance and the intervention and control arms were not statistically significant (e.g., OR 3.02, 95% CI [0.56-16.15] and 2.24, 95% CI [0.33-14.99], for the trust and support scales, respectively, Table 4).

**Table 3:** Results of the regression analysis concerning hypothesis 2.

Variable	Trust in health care workers		Support received by health care workers		Availability of health care workers		Decisional control of patients	
	OR	95% CI	OR	95% CI	OR	95% CI	OR	95% CI
Female (vs. male)	1.14	0.88–1.49	1.02	0.77–1.36	1.12	0.86–1.44	1.07	0.85-1.34
Age 50-69 (vs. 18-49)	1.12	0.77–1.63	1.19	0.80–1.78	1.15	0.79–1.66	0.98	0.71-1.36
Age 70 and older	1.30	0.88–1.93	1.17	0.77–1.79	1.16	0.79–1.70	0.78	0.56-1.10
Low (secondary) education (vs. high)	1.17	0.77–1.77	1.32	0.84–2.05	0.91	0.61–1.36	1.01	0.71-1.45
Vocational educational	1.09	0.74–1.59	1.14	0.76–1.71	1.07	0.74–1.55	1.13	0.80-1.58
Other education	1.42	0.88–2.27	1.89**	1.12–3.20	2.64***	1.62–4.32	1.04	0.69-1.57
Subjective health	1.32***	1.15–1.52	1.32***	1.14–1.53	1.26***	1.10–1.44	1.24***	1.10-1.40
Psychiatric hospital (vs. general hospital)	0.31***	0.19–0.50	0.25***	0.15–0.43	0.35***	0.22–0.58	0.93	0.59-1.47
Length of stay	0.99	0.97–1.01	1.01	0.99–1.03	1.01	0.99–1.03	1.01	0.99-1.02
Intervention ward (vs. control ward)	1.17	0.74–1.83	1.32	0.82–2.12	1.05	0.68–1.61	0.88	0.58-1.32
Survey wave 2020 (vs. 2019)	2.40***	1.54–3.73	2.89***	1.81–4.61	1.65**	1.11–2.47	1.36	0.93-1.98
Survey wave 2021	2.14***	1.46–3.15	3.59***	2.33–5.53	3.09***	2.10–4.55	1.21	0.87-1.69
Intervention ward × 2020 (vs. 2019)	0.65	0.32–1.36	0.77	0.35–1.71	0.86	0.44–1.67	1.03	0.55-1.92
Intervention ward × 2021 (vs. 2019)	0.81	0.42–1.57	0.76	0.36–1.59	1.53	0.76–3.09	1.10	0.63-1.94
Emotional demands	0.78	0.54–1.12	0.92	0.61–1.38	0.55***	0.37–0.80	0.94	0.69-1.28
Cognitive demands	1.31	0.75–2.30	0.81	0.44–1.51	1.22	0.70–2.11	1.50	0.93-2.41
Effort-reward imbalance	1.13	0.51–2.53	1.04	0.43–2.51	0.44*	0.20–0.96	1.33	0.67-2.62
Overcommitment	1.22	0.75–1.98	1.00	0.58–1.71	1.93**	1.19–3.14	0.84	0.57-1.26
Physical work ability	0.84	0.65–1.10	1.00	0.74–1.35	0.71**	0.55–0.91	0.78*	0.62-0.98
Mental work ability	1.00	0.74–1.33	1.12	0.81–1.55	1.02	0.77–1.35	0.87	0.68-1.12

p<0.001 “***”, p<0.01 “**” p<0.05 “*”. OR: odds ratio, 95% CI: 95% confidence interval. N = 1584. Subjective health was treated as a continuous variable with higher scores indicating better health (1 to 5) and odds ratio representing change in the outcomes per one-unit increase in subjective health. Non-significant interaction terms indicate that there is no additional explained variance beyond the main effects.

**Table 4:** Results of the regression analysis concerning the moderation effects of hypothesis 2.

Variable	Trust in health care workers		Support received by health care workers		Availability of health care workers		Decisional control of patients	
	OR	95% CI	OR	95% CI	OR	95% CI	OR	95% CI
Female (vs. male)	1.14	0.88–1.49	1.03	0.77–1.38	1.15	0.88–1.49	1.08	0.86-1.35
Age 50-69 (vs. 18-49)	1.11	0.76–1.61	1.17	0.79–1.76	1.15	0.80–1.66	0.99	0.71-1.38
Age 70 and older	1.32	0.89–1.97	1.21	0.79–1.87	1.23	0.84–1.82	0.82	0.58-1.17
Low (secondary) education (vs. high)	1.16	0.76–1.75	1.33	0.85–2.08	0.89	0.60–1.33	1.02	0.71-1.47
Vocational education	1.07	0.73–1.57	1.13	0.75–1.70	1.06	0.73–1.53	1.13	0.81-1.59
Other education	1.37	0.85–2.20	1.81*	1.07–3.07	2.50***	1.53–4.11	1.04	0.69-1.57
Subjective health	1.32***	1.15–1.51	1.33***	1.14–1.54	1.27***	1.11–1.45	1.26***	1.12-1.41
Psychiatric hospital (vs. general hospital)	0.32***	0.19–0.52	0.26***	0.15–0.44	0.35***	0.21–0.57	0.94	0.59-1.48
Length of stay	0.99	0.97–1.01	1.01	0.99–1.03	1.01	0.99–1.03	1.01	0.99-1.02
Survey wave 2020 (vs. 2019)	2.12***	1.48–3.05	2.76***	1.86–4.07	1.59**	1.13–2.22	1.39*	1.02-1.89
Survey wave 2021	2.08***	1.51–2.88	3.54***	2.45–5.12	3.66***	2.63–5.11	1.29	0.98-1.71
Intervention ward × emotional load	0.86	0.44–1.67	1.20	0.54–2.65	0.62	0.30–1.27	1.54	0.89-2.65
Control ward × emotional load	0.58*	0.34–0.96	0.75	0.43–1.31	0.53**	0.32–0.88	0.74	0.47-1.16
Intervention ward × cognitive load	1.43	0.52–3.90	0.70	0.23–2.13	1.43	0.52–3.95	0.84	0.37-1.93
Control ward × cognitive load	1.60	0.79–3.24	1.02	0.47–2.23	1.25	0.64–2.46	1.74	0.94-3.21
Intervention ward × ERI ratio	3.02	0.56–16.15	2.24	0.33–14.99	0.21	0.03–1.47	0.73	0.18-2.92
Control ward × ERI ratio	0.80	0.31–2.05	0.93	0.33–2.64	0.60	0.25–1.45	1.33	0.60-2.98
Intervention ward × overcommitment	0.83	0.37–1.85	0.90	0.38–2.14	2.41*	1.02–5.73	1.00	0.52-1.93
Control ward × overcommitment	1.57	0.79–3.13	0.92	0.43–1.96	1.45	0.75–2.79	0.82	0.47-1.43
Intervention ward × physical work ability	0.75	0.40–1.40	1.01	0.49–2.08	0.77	0.41–1.47	1.12	0.66-1.89
Control ward × physical work ability	0.86	0.63–1.18	1.07	0.76–1.51	0.76	0.56–1.03	0.78	0.59-1.02
Intervention ward × mental work ability	0.94	0.52–1.70	0.91	0.46–1.80	0.66	0.36–1.19	0.67	0.41-1.09
Control ward × mental work ability	0.97	0.68–1.37	1.09	0.75–1.60	1.08	0.78–1.51	0.88	0.65-1.19

p<0.001 “***”, p<0.01 “**” p<0.05 “*”. OR: odds ratio, 95% CI: 95% confidence interval. N = 1584. Subjective health was treated as a continuous variable with higher scores indicating better health (1 to 5) and odds ratio representing changes in the outcomes per one-unit increase in subjective health. Non-significant interaction terms indicate that there is no additional explained variance beyond the main effects.

## 4 Discussion

The results of the present study did not provide support for the hypothesis that the organisational intervention with health care workers improved patient satisfaction with care regarding trust in health care workers, perceived support and availability of health care workers, and patients’ own decisional control during their hospital stay. There was no indication that the intervention yielded the expected effects, neither directly nor indirectly via the physical and mental work ability of health care workers. In contrast, the largest associations were observed for patient characteristics such as education and subjective health: Patients with primary and secondary education or reporting a better subjective health tended to be higher on all four dimensions of patient satisfaction with care. These results agree well with previous literature in which a lower education and a better subjective health were associated with higher scores of general patient satisfaction (Danielsen et al., 2007, Hekkert et al., 2009, Rahmqvist and Bara, 2010, Koné Péfoyo and Wodchis, 2013).

For the interpretation of the odds ratios reported in the present study, readers should consider the scales of measurement of the variables involved. For example, each unit increase of subjective health was related to an odds ratio of 1.24 concerning the decisional control scale (Table 2). Since subjective health was rated on a 5-point ordinal scale (1: poor health to 5: very good health), the odds ratios may actually vary between OR 1.24 and 2.36 (i.e., poor health vs. very good health, $2.36=\exp^{4\cdot \log\left(1.24\right)}$), respectively. By applying the transformation formula between odds ratios and Cohen’s *d* provided elsewhere ($d=\log\left(OR\right)\cdot \sqrt{3}/\pi$) (Chinn, 2000), the odds ratios of subjective health may represent effect size estimates in the Cohen’s *d* metric between 0.12 and 0.47. Since Cohen’s *d* estimates greater than 0.35 may already be considered medium in social psychology research (Lovakov and Agadullina, 2021), statistically significant odds ratios as low as 1.24 for ordinal scales reported in the present study also comprise medium effect sizes.

The fact that in the present study the lowest scores of patient satisfaction with care were consistently observed among patients in psychiatric hospitals underscores that the assessment of satisfaction with care may be driven mainly by individual appraisal tendencies and interpersonal experiences with health care workers rather than hospital-related characteristics or specific features of the received health care services, in agreement with some previous research findings (Koné Péfoyo and Wodchis, 2013, Batbaatar et al., 2016, Hekkert et al., 2009). This can be concluded, among others, from the fact that a leading symptom of common psychiatric conditions such as social phobia and depression is the tendency to overemphasise negative aspects of situations, personal interactions and events (Peckham et al., 2010, Voncken et al., 2003). Thus, patients’ own judgemental tendencies, attitudes and expectations towards health care services seem to be major determinants of their quality appraisals.

On the other hand, concerning the impact of the psychosocial workload in the wards, the results indicate that patient satisfaction with care as measured in the present study might not be an appropriate indicator of the potential consequences that a high psychosocial workload may exert on the performance of health care workers. To some extent this result follows directly from the observed strong dependence between patient satisfaction with care and patients’ own individual characteristics. As a matter of fact, the observation that the availability scale was more sensitive to changes in emotional demands, increased effort-reward imbalance and health care workers overcommitment in the wards (Table 3) may indicate that only very specific aspects of workers performance, which elicit a more objective judgement of situational features or events (e.g., availability), are more suitable correlates of the psychosocial workload levels and job performance of health care workers than attitudinal or emotion-related indicators (e.g., trust, support). From a broader perspective, these findings are likely to be related to the lack of criterion validity of the scales of patient satisfaction with care which seems to be still pervasive in the literature (Sitzia, 1999, Batbaatar et al., 2015).

Patient satisfaction may be regarded as an equivalent construct to the concept of “customer satisfaction” in marketing (Johnson and Fornell, 1991), which is usually conceptualised as an indicator of perceived performance. Nonetheless, the large ceiling effects found in the present investigation and the lack of criterion validity of the patient satisfaction scales in relation to workers perceived psychosocial workload, work ability and hospital-related characteristics reveals that the causal model implied in Fig. 1 is likely to be ill-defined. For example, there were no associations between work ability and patient satisfaction, with the exception of the unexpected negative association between physical work ability and decisional control (OR 0.71, 95% CI [0.55–0.91] and 0.78, 95% CI [0.62–0.98], Table 3). The lack of sensitiveness of the patient satisfaction scales to patient-independent magnitudes (e.g., working conditions of the health care workers), and their sensitiveness to patient-related features, indicates that the latter are not to be conceived as confounders, but rather as key causal drivers of patients appraisals of health care (see Hall et al. (1993) for a corresponding causal model). As a matter of fact, previous findings suggest that a higher patient satisfaction is associated with increased inpatient utilisation, increased health expenditures and mortality, thereby indicating the limited sensitivity of patient satisfaction scales on quality-process appraisals in health care (Fenton, 2012). By the same token, health care providers should carefully consider the adequacy of the criteria used in quality appraisals of care when evaluating the impact of specific modifications of patient-independent magnitudes. Particular importance should be given to criteria that are highly correlated to the targeted interventions and independent of potential biases resulting from the health status, diagnosis or socio-economic background of patients. On the basis of this study and previous findings mentioned in the introduction, it seems that only very salient aspects of the nursing work environment such as patient-to-nurse ratios, communication skills or rationing policies are more easily perceived by patients and, hence, may correlate more strongly with appraisals of health care. Thus, it can expected that patients will be less able to provide an accurate appraisal of the consequences that may result from complex modifications of the nursing work environment. Therefore, the validity of such appraisals for the purposes of quality control would be much more limited.

### 4.1 Limitations

The results of the present study need to be interpreted against two major limitations. First, the intervention did not specify the exact mechanisms whereby the single measures implemented in the wards may influence patient satisfaction with care, e.g., more patient transport staff or electrocardiogram reading training (Montano et al., 2023). As described in the introduction section, it was simply assumed that any improvement of the physical and mental work ability of workers would increase their performance and, ultimately, result in a higher patient satisfaction with care. The intervention not only lacked the specification of causal mechanisms which may account for the expected outcomes among health care workers (e.g., reduction of the psychosocial workload) (Montano et al., 2023), but also for those among patients (e.g., trust in health care workers). The intervention concept assumed a sort of spill-over effect of potential modifications of the work environment on patient satisfaction, but failed to identify the exact observable magnitudes which would account for the assumed causal associations, e.g., the cognitive processes connecting the observed work environment modifications to the patients’ assessment of their own satisfaction regarding those perceived modifications. Moreover, although the main intervention with health care workers did not contribute to an improvement of their work ability (Montano et al., 2023), the intervention could have resulted in increased patient satisfaction if there were strong mechanisms linking patients’ quality appraisals and specific features of the health care environment. The present analyses could have detected the correlations between the modifications of the work environment and the patient satisfaction scales due to the fact that the workers data were longitudinal. However, the results suggest that these mechanisms were either not identified in the intervention, or are not strong enough to elicit substantial changes in patients’ perceptions of health care services. The second limitation concerns the fact that the patient satisfaction scales showed large ceiling effects, and hence, they were unable to capture the full range of the corresponding constructs (McHorney and Tarlov, 1995), namely, trust, support, availability and decisional control. As already noted decades ago by Sitzia and Wood (Sitzia and Wood, 1997), the patient satisfaction scales are biased in the sense that the scores are usually very high unless a serious adverse event occurs, for example, an incorrect medical treatment (Danielsen et al., 2007).

## 5 Conclusion

In the present study there was no evidence for the hypothesis that an organisational intervention with health care workers had a positive impact on patient satisfaction with care. Furthermore, the associations between the psychosocial workload of workers and the four dimensions of patient satisfaction with care were either contradictory to the expected associations or absent. The results confirmed the observation that individual patient characteristics are the most influential factors of patient satisfaction appraisals and, given the validity shortfall of the concept of patient satisfaction itself, they underline the fact that much more conceptual work needs to be done to understand the mechanisms linking the quality of health care, work process quality, the working conditions of health care workers and patient outcomes.

## CRediT authorship contribution statement

**Diego Montano:** Writing – review & editing, Writing – original draft, Visualization, Software, Methodology, Investigation, Formal analysis, Data curation, Conceptualization.

## Consent to participate

Written informed consent was obtained from the participants prior to participation in the study. All methods were carried out in accordance with relevant guidelines and regulations.

## Ethical considerations

The study was approved by the Ethics Committee of the University of Ulm under the registration number 99/19. The intervention was registered on the German Registry of Clinical Studies (DRKS) with number DRKS00021138.

## Funding statement

The intervention study HALTgeben was funded from 01.02.2019 to 31.01.2022 by the German Federal Innovation Fund under grant agreement 01VSF18006. The funding institution had no influence in the study design, the collection, analysis and interpretation of the data, and the writing of the manuscript.

## Declaration of competing interest

The authors declare the following financial interests/personal relationships which may be considered as potential competing interests: Diego Montano reports financial support was provided by German Innovation Fund. If there are other authors, they declare that they have no known competing financial interests or personal relationships that could have appeared to influence the work reported in this paper.

## Data Availability

The datasets generated and analysed during the current study are not available to third parties due to the German data protection regulations under which this intervention was conducted.
